# The impact of late presentation: analysis of a cohort of 313 Portuguese patients

**DOI:** 10.1186/1758-2652-13-S4-P174

**Published:** 2010-11-08

**Authors:** D Alfaiate, AC Miranda, J Rijo, D Fernandes, F Borges, H Farinha, K Mansinho

**Affiliations:** 1 Hospital de Egas Moniz -CHLO, EPE, Department of Infectious Diseases and Tropical Med, Lisbon, Portugal; 2 Hospital de Egas Moniz -CHLO, EPE, Pharmacy Department, Lisbon, Portugal

## Background

In Europe, 15—38% of HIV infections are diagnosed at advanced stages. Late presentation is associated with increased morbidity and mortality, greater burden for the healthcare system and higher risk of transmission.

## Purpose of the study

To describe the epidemiological and clinical characteristics of a Portuguese cohort of patients diagnosed with HIV infection between 2000 and 2008; to characterize late presenters (LP); to analyse their clinical, immunological and virological evolution and to compare them with non-late presenters (NLP).

## Methods

Retrospective, observational study of patients assisted in an infectious diseases clinic, who were diagnosed with HIV between 01/01/00 and 31/12/08. LP were defined by TCD4+<200/µl or an AIDS defining illness at presentation. SPSS 15.0 was used for statistical analysis.

## Summary of results

Three hundred thirteen patients were included. Most were males (60%) with ages 20-40 years old. About 1/3 (30%) was non-Portuguese. Diagnosis was made by routine serology in 36% of individuals and, in 36%, after development of symptomatic infection. At the time of diagnosis, 42% (n=132) of patients were considered LP. The only risk factor associated with late diagnosis was male gender (p=0,020). Average TDC4 count at baseline was 132/µl for LP and 497/µl for NLP (p=0,0006). Combined antiretroviral therapy (cART) was started in 100% LP vs 74% NLP (p<0,0001), with NNTRI being the most frequent regimen (non significant). Both groups showed an increase in TCD4 counts over time (average increase of 366/µl LP vs 121/µl NLP; p<0,0001). At present time, 87% LP have undetectable plasma HIV RNA vs 79% NLP (non significant) and average TCD4 counts are 495/ µl for LP vs 610/ µl for NLP (p=0,0006). LP had more hospital admissions (51% vs 22% in NLP; p<0,0001), most of which AIDS related (LP 52% vs 15% for NLP; p<0,0001. Most patients in both groups remained adherent to regular medical follow-up and there was no significant difference in the mortality rate.

## Conclusions

LP represented a significant proportion of HIV diagnosed patients and were associated with more AIDS related events and hospital admissions, but not higher mortality rate. Prompt institution of cART allowed a significant immune recovery, although not enough to match NLP CD4 counts. These data support the need for effective screening strategies in order to allow an earlier diagnosis of HIV infection and a better long term prognosis.

